# Current and future therapeutic strategies for high-grade gliomas leveraging the interplay between epigenetic regulators and kinase signaling networks

**DOI:** 10.1186/s13046-023-02923-7

**Published:** 2024-01-05

**Authors:** Lea M. Stitzlein, Jack T. Adams, Erin N. Stitzlein, Richard W. Dudley, Joya Chandra

**Affiliations:** 1 Department of Pediatrics Research, The MD Anderson Cancer Center, University of Texas, Box 853, 1515 Holcombe Blvd, Houston, TX 77030 USA; 2 The University of Texas MD Anderson Cancer Center UTHealth Graduate School of Biomedical Sciences, Houston, TX USA; 3 Drexel University College of Medicine, Philadelphia, PA USA; 4 Department of Pharmaceutical Sciences, University of Findlay, Findlay, OH USA; 5 Department of Epigenetics and Molecular Carcinogenesis, The MD Anderson Cancer Center, Houston, TX USA

**Keywords:** High-grade gliomas, Glioblastoma, Diffuse midline gliomas, Epigenetic regulators, Epigenetically directed therapy, Targeted therapy

## Abstract

Targeted therapies, including small molecule inhibitors directed against aberrant kinase signaling and chromatin regulators, are emerging treatment options for high-grade gliomas (HGG). However, when translating these inhibitors into the clinic, their efficacy is generally limited to partial and transient responses. Recent studies in models of high-grade gliomas reveal a convergence of epigenetic regulators and kinase signaling networks that often cooperate to promote malignant properties and drug resistance. This review examines the interplay between five well-characterized groups of chromatin regulators, including the histone deacetylase (HDAC) family, bromodomain and extraterminal (BET)-containing proteins, protein arginine methyltransferase (PRMT) family, Enhancer of zeste homolog 2 (EZH2), and lysine-specific demethylase 1 (LSD1), and various signaling pathways essential for cancer cell growth and progression. These specific epigenetic regulators were chosen for review due to their targetability via pharmacological intervention and clinical relevance. Several studies have demonstrated improved efficacy from the dual inhibition of the epigenetic regulators and signaling kinases. Overall, the interactions between epigenetic regulators and kinase signaling pathways are likely influenced by several factors, including individual glioma subtypes, preexisting mutations, and overlapping/interdependent functions of the chromatin regulators. The insights gained by understanding how the genome and epigenome cooperate in high-grade gliomas will guide the design of future therapeutic strategies that utilize dual inhibition with improved efficacy and overall survival.

## Introduction

High-grade gliomas (HGGs) are central nervous system (CNS) tumors that occur in both children and adults, although bear distinct molecular features and neuroanatomy in younger compared with older patients [1–4]. While these types of cancers are relatively rare, patient prognosis is quite poor, with an average 2-year overall survival of only 20% [5], despite multimodal treatment regimens consisting of surgery, radiation therapy, and chemotherapy [6, 7]. Tumor location or disease progression can further complicate therapeutic interventions; therefore, novel treatment modalities such as targeted therapies, including epigenetically directed therapies, are critical to improve patient outcomes.

The identification of cancer-specific targets supporting tumor growth is a major research objective to develop therapeutic options in HGG. Advances in sequencing technology and single-cell analyses of HGGs and subsets of medulloblastoma have revealed frequent alterations in kinase signaling proteins and proteins which regulate their activity (i.e., tumor suppressors) [8–11]. For example, several receptor tyrosine kinases (RTKs) are frequently overexpressed or mutated in HGG [8, 11], causing hyperactivity of downstream signaling cascades leading to increased cell proliferation, growth, and survival of cancer cells. To date, there has been limited clinical efficacy from single-agent inhibition of dysregulated signaling pathways in HGG [12].

In addition to aberrant proliferative signaling pathways, disruption of the epigenome has been identified as a contributor to tumorigenesis, cancer progression, and chemotherapy resistance [13, 14]. Epigenetic regulators, termed “writers” and “readers”, catalyze the reversible chemical modifications of histones and DNA [15]. The most predominant epigenetic alterations are post-translational modifications to histones, involving the addition and removal of methyl and acetyl marks, and DNA methylation [16]. The epigenetic “readers” recognize specific modifications and translate their effects on gene expression and other cellular processes. Pharmacological inhibitors have been developed to target various chromatin regulators, such as those directed against the histone deacetylase (HDAC) family, bromodomain and extraterminal (BET)-containing proteins, protein arginine methyltransferase (PRMT) family, Enhancer of zeste homolog 2 (EZH2), and lysine-specific demethylase 1 (LSD1).

Understanding the complex interactions between the cancer genome and epigenome is paramount when designing novel therapeutic strategies. Targeted therapies directed solely at epigenetic regulators or dysregulated kinases have shown limited success in sustaining clinical responses in HGG [17–22]. Evidence increasingly shows that epigenetic modulators cooperate with several relevant kinase signaling pathways in gliomas to promote cancer progression and contribute to therapeutic resistance. In this review, we systematically explore the epigenetic regulators and their interactions with kinase signaling networks in HGG and how combination strategies have developed and could be envisioned via existing small molecule inhibitors.

## Molecular dysfunction of the genome and epigenome

### Kinase signaling pathway alterations

Over the past two decades, substantial effort has been made to sequence and molecularly characterize primary cancer samples across all cancer types, including adult and pediatric HGG. This initiative has identified frequent alterations in several kinase signaling pathways and their regulators (Table 1.) One of the more common alterations detected involve the RTKs, including epidermal growth factor receptor (*EGFR*), platelet-derived growth factor receptor (*PDGFRA*), and *KIT*, also known as mast/stem cell growth factor receptor. These alterations are composed of gene mutations and/or copy number amplification, which hyperactivate downstream signaling networks involved in cell proliferation, differentiation, cell growth, metabolism, and survival. One convergent activating pathway downstream of these RTKs is phosphatidylinositol-3 kinase (PI3K)/AKT/mammalian target of rapamycin (mTOR) signaling. Alterations are found in the catalytic subunit of PI3K, *PIK3CA*, and the regulatory subunit, *PIK3R1*, which can activate downstream signaling at AKT and mTOR. Additionally, copy number deletions are found within Phosphatase and Tensin Homolog (*PTEN*), a tumor suppressor that negatively regulates PI3K/AKT signaling. A common deletion in *PTEN* includes homozygous deletion, which contributes to hyperactivation of the PI3K/AKT/mTOR pathway.

In addition to the PI3K/AKT pathway, the mitogen-activated protein kinase (MAPK) cascade is downstream of RTKs and involved in HGGs. In this pathway, loss of function mutation in neurofibromin 1 (*NF1*), a small GTPase activating protein that regulates signal transduction through RAS (Rat sarcoma virus), affects the downstream MAPK signaling leading to activation. Lastly, the cell cycle control gene, cyclin-dependent kinase inhibitor 2 A (*CDKN2A*), is frequently found to have homozygous deletion. Beyond the aforementioned kinase alterations, several other gene alterations form the HGG genomic landscape. When comparing adult and pediatric gene alterations, there are many overlapping changes within the kinase signaling pathways; however, the frequency at which they occur differs with age.

**Table 1:** Alterations within kinase signaling cascades and their regulators across adult and pediatric high-grade gliomas [23, 24]. The frequency of gene mutations and copy number alterations were obtained from the cBioPortal for Cancer Genomics using two data sets, Glioblastoma Multiforme (TCGA, Firehose Legacy) and Pediatric Brain Cancer (CPTAC/CHOP, Cell 2020). AMP – amplification; CNA – copy number alterations; HOMDEL – homozygous deletion; pHGG – pediatric high-grade glioma

Gene	Cancer Type	Frequency of mutation	Frequency of CNA
*EGFR*	Glioblastoma	26.6%	43.8% (AMP)
	pHGG	4%	5% (AMP)
*PTEN*	Glioblastoma	31.0%	9.7% (HOMDEL)
	pHGG	–	5% (HOMDEL)
*PIK3CA*	Glioblastoma	11.0%	2.8% (AMP)
	pHGG	8.0%	–
*PIK3R1*	Glioblastoma	11.4%	0.3% (HOMDEL)
*NF1*	Glioblastoma	11%	1.9% (HOMDEL)
	pHGG	24%	–
*CDKN2A*	Glioblastoma	0.7%	57.4% (HOMDEL)
	pHGG	–	35.0% (HOMDEL)
*KIT*	Glioblastoma	1.0%	9.2% (AMP)
	pHGG	–	15% (AMP)
*PDGFRA*	Glioblastoma	3.8%	12.8% (AMP)
	pHGG	8.0%	10% (AMP)

### Epigenetic abnormalities from histone modifiers

Unlike genetic changes, epigenetic dysregulation is not typically the result of mutations and instead occurs through alterations in chromatin accessibility and gene expression [25]. Transcriptional dysregulation can result from overexpression of chromatin modulators and their subsequent hyperactivity. The absence or presence of specific histone modifications, including acetylation and methylation of critical amino acids, governs chromatin structure leading to changes in gene transcription.

Numerous enzymes and protein complexes have been identified to be responsible for regulating the expression of various genes. These epigenetic proteins are over- or under-expressed in tumors, including HGG. One of the most well-studied epigenetic modulators is the HDAC family of enzymes. In gliomas, HDAC activity generally suppresses the expression of regulatory proteins and DNA repair genes as a component of repressive transcriptional complexes. Several HDAC family members have been identified as having altered gene expression in HGG. For example, HDAC1, 2, 3, and 7 have been found to be overexpressed in grade IV gliomas compared to normal brain tissue and low-grade gliomas [26]. Meanwhile, other enzymes that act as readers of these acetylated amino acids are often dysregulated in gliomas. These sets of proteins include the BET proteins. Two BET proteins, BRD2 and BRD4, are significantly overexpressed in gliomas, and the knockout of BRD4 diminishes glioma proliferation [27]. Similarly, PRMT enzymes, such as PRMT1, 2, and 5, function to methylate arginine residues and can promote dysregulation in brain tumors arising from the aberrant expression or activity [28–31]. Another epigenetic regulator relevant to HGG is the methyltransferase, EZH2, which is overexpressed in gliomas and correlated with high-grade gliomas [32, 33]. EZH2’s activity contributes to glioma progression by silencing tumor-suppressor genes [34]. An additional epigenetic modulator that is commonly dysregulated in gliomas is LSD1, a histone demethylase that is a component of several repressive complexes and is associated with reduced gene transcription. LSD1 is found to be overexpressed in several cancer types, including glioblastoma [35–37], and has been associated with poor patient prognosis in certain types of cancer [38–42]. Understanding the dysregulation of epigenetic modulators in HGG can inform the development of targeted therapies aimed at restoring normal gene expression and halting tumor growth.

The aforementioned epigenetic modulators are found in large epigenetic protein complexes, often with several epigenetic proteins. The complexes serve to activate its members and increase their stability. One of the most well-known epigenetic protein complexes is the CoREST (REST corepressor 1), which functions to enhance nucleosome regulation and drive gene repression [43–46]. The CoREST complex includes both HDAC1/2 proteins and LSD1. Another complex associated with LSD1 is the nucleosome remodeling and deacetylase (NuRD) complex, also including HDAC1/2 proteins [47]. An important role the NuRD complex plays is to maintain the genomic landscape and regulate cell cycle progression [48]. Similarly, the polycomb repressive complex (PRC2) is a repressive chromatin complex relevant to HGG and includes EZH2. This complex functions to regulate normal embryonic development and proper cell identity [49]. Finally, PRMT5 is a part of the methylosome, a protein complex that functions to methylate arginine residues of spliceosomal proteins important for the assembly of small nuclear ribonucleoproteins [50].

Overall, the activity of chromatin modulators and their histone modification can prompt severe changes in gene transcription that can lead to an oncogenic phenotype (Table 2). Their effects on gene transcription can impact various biological processes, from cell division and proliferation to differentiation. Largely, oncogenic conversion via epigenetic regulation can be driven by the actions of tumor suppressor proteins and signaling kinases, highlighting the potential interplay of kinase activity and epigenetic intervention.

**Table 2:** Dysregulation of gene transcription resulting from the activity of chromatin regulators in adult and pediatric high-grade gliomas. Epigenetic dysregulation arises from changes in histone acetylation and methylation through changes in protein expression and activity to affect gene transcription. BET – bromodomain and extraterminal; EZH2 – enhancer of zeste homolog 2; HDAC – histone deacetylase; H3K4/9/27 – histone 3 lysine 4/9/27; LSD1 – lysine-specific demethylase 1; PRMT – protein arginine methyltransferase (PRMT) family

Epigenetic modification	Protein(s)	Function	Effect on gene transcription	Implications in HGG
Histone acetylation	HDAC family	Acetyl eraser	Remove acetyl groups at H3K4 to downregulate gene expression	Overexpression of HDAC 1/2/3/7 [26]
	BET family	Acetyl reader	Recognize acetylated lysine residues to activate or repress transcription	Overexpression of BRD2 and BRD4 [27]
Histone methylation	PRMT family	Methyl writer	Dual activity as a transcriptional activator and repressor depending on histone mark subject to arginine methylation	PRMT 1/2/5/3/6 upregulated or overexpressed in HGG [28–31, 51, 52]
	EZH2	Methyl writer	Transcriptional repression via hypermethylation of histone H3K27	Overexpression in gliomas is associated with poor prognosis [32, 33]
	LSD1	Methyl eraser	Demethylate lysine residues on H3K4 to repress gene transcription and on H3K9 to activate gene transcription	Overexpressed in glioblastoma [35–37]

### Altered DNA methylation patterns

DNA methylation, like histone modifications, is involved in the regulation of gene expression, typically to repress gene transcription [16]. The addition of methyl groups to DNA most commonly occurs at cytosine residues to form 5-methylcytosine in CpG sites, where cytosine is linked to a guanine nucleotide by a phosphate group [16]. The enzymes responsible for generating 5-methylcytosine at the CpG sites are the DNA methyltransferases (DNMTs). The DNMT family includes DNMT1, DNMT3A, and DNMT3B, which establish and maintain the pattern of DNA methylation. The de novo methyltransferases, DNMT3A and DNMT3B, initiate the CpG methylation pattern. Meanwhile, DNMT1 is a maintenance methyltransferase and retains methylation marks throughout DNA replication and cell division.

In gliomas, DNA methylation patterns are used alongside histopathology for tumor classification, glioma subtyping, and as a prognostic biomarker [53]. For example, methylation profiling can be used as a surrogate to identify the mutation status of isocitrate dehydrogenase (IDH) [53]. IDH mutations, associated with the production of an oncometabolite (2-hydroxyglutarate), leads to global hypermethylation of the CpG islands, thereby causing gene silencing [54, 55]. The presence of this methylation pattern in gliomas is called the glioma – CpG island methylator phenotype (G-CIMP) and occurs frequently in low-grade gliomas [54, 55]. Large cohort studies have shown that G-CIMP is highly associated with the presence of an IDH mutation and correlated with a favorable prognosis [56]. Similarly, histone mutations common to pediatric HGGs lead to global changes in DNA methylation that can be detected through DNA methylation profiling [55, 57]. Methylation profiling can be used to derive copy number profiles inclusive of gene amplifications/deletions and chromosome alterations (7+/10- and 1p/19q codeletion) associated with different glioma subtypes [53]. Finally, the DNA methylation status of *O6-methylguanine-DNA methyltransferase* (*MGMT*) is widely used as a predictive biomarker for therapeutic response to the alkylating agent, temozolomide, and as a prognostic marker in glioblastoma patients [55, 58]. Thus, the value of methylation profiling in gliomas is expanding beyond its impact on gene expression, to help determine tumor phenotype and prognosis.

The World Health Organization (WHO) is beginning to adopt DNA methylation profiling in their classification of CNS tumors to provide a robust classification method and identify new tumor types and subtypes [53]. Notably, the emergence of DNA methylation profiling reveals an intriguing overlap with distinct kinase mutations traditionally associated with gliomas. For example, through methylation profiling, HGG is grouped into eight classes, including three classes characterized by RTK alterations, such as *PDGFRA* and *EGFR* amplification [59]. Furthermore, in pediatric HGGs with histone alterations, methylation profiling revealed several alterations in kinase signaling pathways, including *PDGFRA*, *EGFR*, *KIT*, *MET*, *KRAS*, *PTEN*, *PIK3CA*, and *CDK4/6* [57]. Additional studies of pediatric HGGs used DNA methylation alongside whole genome sequencing and RNA sequencing in a clinical trial to molecularly characterize tumors and determine a treatment approach based on the identified alterations [60]. Overall, these studies highlight the potential of methylation profiling and its utility in understanding glioma subtypes, their associated kinase alterations, and appropriate therapeutic selection.

## Single-agent targeted therapies in clinical development

### Kinase inhibitors

Clinical trials have been implemented and are currently underway to assess the safety and efficacy of small molecule inhibitors directed against protein or lipid kinases in HGG patients with kinase dysregulation and aberrant signaling activation (Table 3). Several clinical studies have tested the effects of inhibition of RTKs, including small molecule inhibition of EGFR, PDGFR, FGFR (fibroblast growth factor receptor), c-MET (mesenchymal-epithelial transition factor), KIT (also referred to as CD117), AXL, and VEGFR (vascular endothelial growth factor receptor). Numerous inhibitors have assessed inhibition of EGFR in HGG as single agents and predominantly show a tolerable safety profile without improvements in survival. Likewise, the results from available clinical trials targeting other RTKs, PDGFR and MET, show tolerable safety profiles but limited efficacy. Currently, other RTK inhibitors targeting FGFR and KIT are being investigated in early-phase trials to determine the safety and efficacy of these small molecule inhibitors for adult (FGFR and AXL) and pediatric HGGs (KIT). Studies are also investigating the inhibition of intracellular signaling kinases in HGG downstream of RTKs. For example, clinical studies are ongoing in pediatric HGG patients receiving small molecule inhibitors against MEK and PI3K. Overall, many completed clinical trials conclude that single-agent inhibitors have tolerable toxicities in phase I but limited efficacy when assessed in phase II trials. This lack of clinical efficacy has been extensively reviewed elsewhere [61–64]. To summarize, resistance can occur from downstream activation of nontargeted kinase pathways or activation of parallel signaling pathways. Other resistance mechanisms, or perhaps a limited tumor penetrance, may be at play, underscoring the lack of response in these clinical trials. Heterogeneity among the brain tumor cells may also prevent the various single-agent strategies utilizing kinase inhibitors from producing efficacious results in reducing tumor burden or prolonging survival.

**Table 3:** Previous and ongoing clinical trials inclusive of single-agent treatments directed against protein and lipid kinases in high-grade gliomas. DIPG – diffuse intrinsic pontine glioma; DLT – Dose-limiting toxicity; EGFR – epidermal growth factor receptor; ERK – extracellular signal-regulated kinase; FGFR – fibroblast growth factor receptor; GBM – glioblastoma; HGG – high-grade glioma; MAPK – mitogen-activated protein kinase; MEK – mitogen-activated protein kinase kinase; MET – mesenchymal-epithelial transition factor; MTD – maximum tolerated dose; PD – pharmacodynamic; PDGFR – platelet-derived growth factor receptor; PFS – progression-free survival; pHGG – pediatric high-grade gliomas; PI3K – phosphatidylinositol-3 kinase; PK – pharmacokinetic; RT – radiation therapy; VEGFR – vascular endothelial growth factor

Target	Intervention	Condition	Phase	Outcome of the primary endpoint	
EGFR	Erlotinib	Recurrent EGFRvIII GBM	I	Clinical benefit rate, no results yet (NCT01257594)	
		Pediatric recurrent brain tumors and brain stem glioma [65]	I	Tolerable safety profile in pediatric patients (NCT00418327)	
		Recurrent GBM [66]	I/II	High toxicity and minimal benefit as monotherapy (NCT00301418)	
		Young patients with newly diagnosed GBM [67]	I/II	Tolerable safety profile but poor efficacy (NCT00124657)	
		Recurrent GBM [68]	II	Insufficient single-agent activity and no improvement in survival (NCT00086879)	
		Recurrent GBM	II	Disease progression in all patients, study terminated (NCT00387894)	
		Recurrent or progressive GBM	II	Response rate, no results yet (NCT00054496)	
	Gefitinib	Adult GBM [69]	I/II	Well tolerated when combined with RT, but median survival is similar to RT alone (NCT00052208)	
		Newly diagnosed pediatric brain stem gliomas [70]	I/II	Tolerable safety profile (NCT00042991)	
		Newly diagnosed GBM [71]	II	No improvement in overall survival (NCT00014170)	
		Recurrent GBM after standard treatment [72]	II	Dephosphorylates EGFR without affecting downstream signaling (NCT00250887)	
	Afatinib	Recurrent GBM	I/II	Tolerable safety profile but limited single-agent efficacy (NCT00727506)	
	Lapatinib (EGFR/HER2)	Recurrent HGG	I	Determine PK and PD parameters, no results yet (NCT02101905)	
	Dacomitinib	Recurrent GBM with EGFR amplification or EGFRvIII [73]	II	Limited single-agent activity (NCT01520870)	
	Epitinib	GBM	I	Objective response rate, no results yet (NCT03231501)	
	Tesevatinib	Recurrent GBM	II	Limited single-agent activity (NCT02844439)	
PI3K	Paxalisib	pHGG	I	Determine the MTD, adverse effects, and pharmacokinetic profile, no results yet (NCT03696355)	
		Newly diagnosed GBM	II	Determine DLTs, no results yet (NCT03522298)	
MEK	Trametinib	Pediatric gliomas with MAPK/ERK activation	II	Objective response rate, no results yet (NCT03363217)	
ERK	Ulixertinib	Advanced solid tumors with MAPK pathway mutations, including recurrent/refractory gliomas	II	Objective response rate, no results yet (NCT03698994, NCT03155620, NCT02465060, NCT04566393)	
FGFR	Pemigatinib	Recurrent GBM with activating FGFR1-3 alterations	II	Objective response rate, no results yet (NCT05267106)	
	Infigratinib	Recurrent GBM with FGFR1-3 alterations [20]	II	16% PFS at 6 months (limited efficacy, but disease control in a subset of patients with FGFR1/3 point mutations and FGFR3-TACC3 fusions) (NCT01975701)	
		Recurrent HGG with FGFR1/3 point mutations or FGFR3-TACC3 translocation	I	Determine the PK parameters and 6-month PFS for expansion cohort, no results yet (NCT04424966)	
	Erdafitinib	Advanced solid tumors with FGFR mutations, including relapsed malignant gliomas	II	Response rate, no results yet (NCT03210714); objective response rate, no results yet (MATCH trial: NCT02465060)	
PDGFR	Crenolanib	DIPG or recurrent pHGG [21]	I	Toxicity profile similar to adults, but no preliminary signs of efficacy (NCT01393912)	
		Recurrent GBM with PDGFR alteration	II	PFS at 6 months, no results yet (NCT02626364)	
	Avapritinib (KIT/PDGFRA)	Pediatric solid tumors with KIT/PDGFRA alteration	I/II	Determine dose for phase II and overall response rate, no results yet (NCT04773782)	
MET	Bozitinib (PLB-1001)	Recurrent glioma with MET fusion [22]	I	Tolerable safety profile and partial response in two patients (NCT02978261)	
	Vebreltinib (APL-101)	Advanced solid tumors, including GBM	I/II	Determine the MTD, DLT, and objective response rate, no results yet (NCT03175224)	
KIT	DCC-2618	Advanced malignancies, including glioma	I	Determine safety, MTD, ORR, and DCR, no results yet (NCT02571036)	
VEGFR	Anlotinib (multi-targeted agent)	Recurrent GBM/HGG [74]	I/II	PFS, no results yet (NCT04004975; NCT04822805)	
AXL	Bemcentinib (BGB324)	Recurrent GBM undergoing surgery	I	Determine the PK and PD parameters, no results yet (NCT03965494)	

### Epigenetically directed therapies

Targetable epigenetic regulators are being investigated in HGGs as single-agent therapies (Table 4). Similar to kinase inhibition, available results from clinical trials lend tolerable safety profiles. To date, there is limited information on the clinical benefit of these drugs compared to the standard of care. So far, pharmacological HDAC inhibition via vorinostat has been well tolerated and has provided a modest improvement in progression-free survival (PFS) [75]. Other HDAC inhibitors, panobinostat and an aqueous formulation of panobinostat (MTX110), have been assessed in a population of diffuse midline gliomas (DMG, previously referred to as DIPG) and show an acceptable safety profile [19, 76]. However, at tolerable doses panobinostat has no significant clinical benefit likely due to lack of drug exposure at the tumor site [19]. Fortunately, the administration of MTX110 via convection-enhanced delivery provided encouraging results with a benefit on overall survival compared with historical outcomes [76]. The positive results from MTX110 on survival will be assessed further in a multicenter phase II study. In contrast, the clinical studies of birabresib, a BET inhibitor, in HGGs showed a lack of clinical efficacy [18]. Perhaps the insufficient activity is due to intra-tumor heterogeneity and the emergence of subpopulations resistant to treatment. Lack of brain penetrance seems unlikely as preclinical pharmacokinetic analysis indicated biologically active levels of birabresib [77]. Altogether, the outcome of these clinical trials justifies a need to understand further mechanisms of resistance and ways to enhance their efficacy. One potential approach is to investigate the interplay with proliferative kinase signaling pathways.

**Table 4:** Clinical trials of epigenetically directed therapies in adult and pediatric high-grade gliomas from the past and ongoing. CNS – central nervous system; DIPG – diffuse intrinsic pontine gliomas; DLT – dose-limiting toxicity; GBM – glioblastoma; MTD – maximum tolerated dose; OS – overall survival; PFS – progression-free survival

Target	Intervention	Condition	Phase	Outcome of the primary endpoint	Response Biomarker
HDAC family	Vorinostat	Progressive or recurrent GBM [75]	II	Well tolerated and modest improvement in PFS (NCT00238303)	Acetylated H2BK5, H3K9, and H4K8
	Entinostat	Pediatric patients with solid tumors, including the CNS [78]	I	Tolerable safety profile and no DLTs (NCT02780804)	Global protein lysine acetylation
	Panobinostat MTX110 (Aqueous panobinostat)	DIPG [19] DIPG [76)	I I/II	The MTD is 10mg/m^2^/dose for progressive DIPG and 22mg/m^2^/dose for pre-progressive DIPG when given 3x/week for 3 weeks on/1 week off. DLTs include thrombocytopenia and neutropenia (NCT02717455) Tolerable safely profile and OS of 26.1 months (NCT03566199)	Not included
BET family	Birabresib	Recurrent GBM	II	Study terminated due to a lack of clinical activity (NCT02296476)	Not included
PRMT5	PRT811	Advanced solid tumors, including high-grade gliomas	I	Toxicity profile and DLT, no results yet (NCT04089449)	No results yet
EZH2	Tazemetostat	Pediatric patients with relapsed/refractory solid tumors with EZH2 alteration	II	Objective response rate, no results yet (NCT03155620; NCT03213665)	No results yet

## Crosstalk from epigenetic modulators of histone acetylation with kinase signaling

### Histone deacetylases (HDAC)

**Fig. 1:**
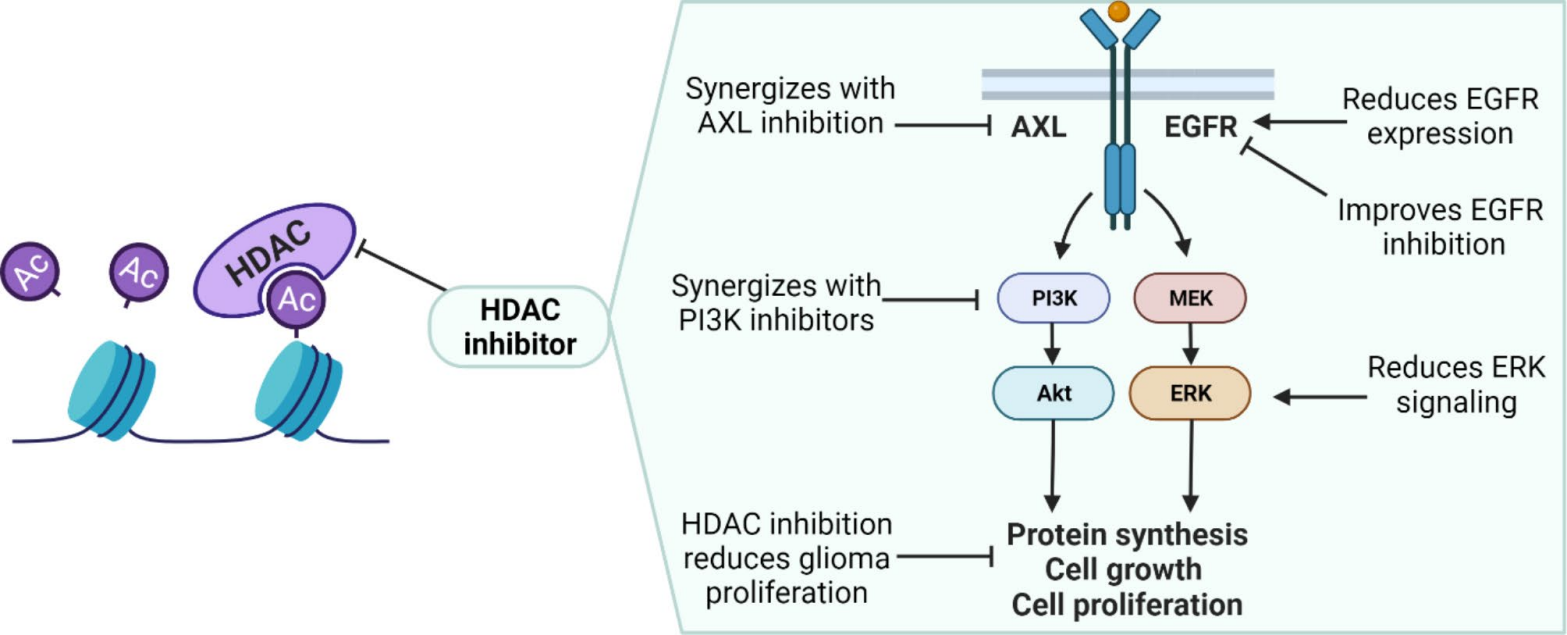
Modulation of Kinase Signaling Pathways by HDAC Inhibitors. HDAC inhibitors exhibit synergistic interactions with EGFR and AXL inhibitors, enhancing their therapeutic potential [92]. Additionally, HDAC inhibitors downregulate EGFR expression and attenuate ERK signaling, potentially disrupting downstream cascades [87, 93, 95]. HDAC inhibitors also synergize with PI3K inhibitors, leading to a reduction in phospho-AKT signaling [83]. These observations highlight the intricate regulatory role of HDAC inhibitors in modulating diverse aspects of kinase signaling networks for potential therapeutic applications Created by BioRender.com

Histone deacetylases (HDACs) are a large family of enzymes that catalyze the removal of acetyl groups from lysine residues on histone tails and non-histone targets; this action generally limits chromatin accessibility and reduces gene transcription. Eighteen human HDACs are separated into four groups based on homology [79]. While HDACs are responsible for deacetylation, they oppose histone acetyltransferase (HATs), which catalyzes reversible lysine acetylation that results in a more permissive gene expression. Therefore, the balance in HDAC and HAT activity is vital for the proper and timely repression or expression of genes. Inhibitors of HDAC have progressed into the clinic, receiving FDA approval for the treatment of several hematological malignancies. To date, the clinically approved HDAC inhibitors (vorinostat, romidepsin, panobinostat, and belinostat) are known as pan-HDAC inhibitors and act on multiple classes of HDACs. In cancer treatment, HDAC inhibitors are used to induce cell cycle arrest and cell death by increasing gene transcription, such genes involved in both intrinsic, *BAX, BAK, APAF1*, and extrinsic, *TRAIL, DR5, FAS, FAS-L*, and *TNF-α*, apoptotic pathways [80]. Currently, HDAC inhibitors are being assessed in glioblastoma, pediatric HGG, and medulloblastoma models. HDACs have been suggested to regulate glioma proliferation via interactions with the PI3K/AKT signaling pathway, MAPK signaling, and upstream at receptor tyrosine kinases (Fig. 1).

In various cancer models, HDAC antagonists have a synergistic relationship with PI3K inhibitors that is associated with a strong inhibition of the PI3K/AKT signaling pathway compared to single-agent PI3K inhibitor [81, 82]. In GBM cell models, panobinostat has been shown to synergize with a PI3K/mTOR inhibitor to reduce cell viability [83]. In this study, a dual inhibitor of PI3K and mTOR, dactolisib, was used in combination with panobinostat: their effects on cell viability, cell proliferation, and induction of apoptosis were assessed. The combination treatment resulted in a synergistic reduction in cell viability, enhanced antiproliferative effects, and induced apoptosis relative to single-agent treatment [83]. Furthermore, the combination treatment enhanced AKT signaling reduction compared to the single-agent treatment [83].

Similar to adult GBM, a study found that inhibition of HDAC/PI3K via dual inhibitor, CUDC-907, in pediatric HGG models exerts substantial antitumor effects compared to single-target inhibition [84]. CUDC-907 has been shown to reduce the PI3K signaling network and, importantly, blocks compensatory signaling via MAPK and STAT3 (signal transducer and activator of transcription) signaling pathways [85]. Interestingly, this dual inhibitor acts as a radiosensitizer in pediatric HGG models mediated by decreasing NFκB/Forkhead box M1 (FOXM1) expression [84]; this additional effect is particularly important in the pediatric HGG and DMG, as radiation therapy is the current standard of care. Identifying novel strategies to enhance radiotherapy efficacy will be important when progressing into clinical trials. One additional study investigated the HDAC inhibitor, panobinostat, in combination with PI3K inhibition in medulloblastoma. This study showed that HDAC inhibition combined with PI3K inhibition synergistically inhibited growth by activating a tumor suppressor, FOXO1 via two distinct mechanisms [86]. In this case, HDAC inhibition increased FOXO1 protein expression, associated with increased acetylation of H3K9 and H3K27, while PI3K inhibition promoted nuclear localization of FOXO1 through its dephosphorylation [86]. Overall, multiple studies have examined the co-inhibition of HDACs and PI3K/AKT signaling networks and observed enhanced efficacy compared to HDAC inhibition alone.

Apart from the PI3K/AKT signaling pathway, HDAC inhibitors cooperate with several members of the MAPK family proteins. For example, the HDAC inhibitor, sodium butyrate, markedly reduces ERK (extracellular signal-regulated kinase) activation in medulloblastoma with increased H3 acetylation [87]. From this study, the dual inhibition of HDAC and MAPK signaling reduces medulloblastoma proliferation and viability. C-Jun N-terminal kinase (JNK), another member of the MAPK superfamily, can be activated in gliomas and is crucial in the maintenance of stemness [88, 89]. HDAC6 promotes cell growth in glioblastoma through the JNK signaling pathway [90]. Furthermore, inhibition of HDAC6 by ricolinostat, associated with increased H3K9 and H3K27 acetylation, suppresses JNK activity and mediates reduced proliferation and invasion of glioma cells [90]. An additional regulator of MAPK signal transduction is the MAPK phosphatase, MKP1. MKP1 is an inhibitor of ERK1/2, JNK, and MAPK to regulate glioma self-renewal and differentiation. Interestingly, upregulation of MKP1 occurs following treatment with the HDAC inhibitor vorinostat and further sensitizes glioblastoma cells to temozolomide [91]. Finally, the inhibition of RTKs, located upstream of PI3K/AKT and MAPK signaling pathways, has also been shown to improve anti-tumor activity in combination with HDAC inhibitors to target HGG. A study in DMG found that the RTK, AXL, is upregulated in DMG, initiates the mesenchymal transition, and dual AXL/HDAC inhibition caused a synergistic anti-tumor effect [92]. The treatment combination of BGB324 (AXL inhibitor) and panobinostat causes the downregulation of genes associated with mesenchymal transition and DNA damage repair, ultimately leading to decreases in cell viability [92]. Understanding the interplay between HDAC activity and RTK/MAPK signaling pathways is valuable to establish the comprehensive mechanism by which HDAC inhibitors act in the context of HGG.

Beyond AXL, HDAC inhibitors have shown synergistic relationship with other RTKs, including EGFR. Two recent studies assessed the effects of dual inhibition of HDAC and EGFR. In glioblastoma cells, combining EGFR inhibition via AG1478 with sodium butyrate, resulted in decreased cell viability with more activity than either agent alone [93]. Interestingly, the combination mentioned previously increased STAT3 mRNA expression [93]. The authors speculate that the upregulation of STAT3 may mediate the anti-tumor effects of dual HDAC/EGFR inhibition through a tumor-suppressive role, as opposed to its dual action as an oncoprotein [93, 94]. Furthermore, another study also combined EGFR and HDAC inhibitors to study their effects on glioblastoma cells with various models of EGFR alterations [95]. The combined effects of erlotinib and investigational HDAC inhibitor, scriptaid, increased H3K9 acetylation and could overcome erlotinib resistance and re-sensitize glioblastoma cells to EGFR inhibition [95]. In fact, the combination enhanced single-agent efficacy in glioblastoma cells independent of their EGFR status. Further experiments were completed to understand this relationship of enhanced efficacy; HDAC inhibition, via vorinostat or scriptaid, caused a decrease in the mRNA and protein expression of EGFR, both wild-type and EGFRvIII (EGFR variant III extracellular domain mutation) [95]. Due to promising results, the combination of EGFR and HDAC inhibition has been assessed in a phase I/II clinical trial in recurrent glioblastoma (NCT01110876). Unfortunately, this study was terminated due to unanticipated toxicities before there could be any assessment of efficacy in the phase II portion of the trial. While the combination had unacceptable toxicities, their EGFR inhibitor, erlotinib, has limited ability to penetrate the blood-brain-barrier (BBB) and is only active against wild-type EGFR. Future combination studies could include EGFR inhibitors with high penetrance across the BBB as well as a focus on the development of EGFR inhibitors selective for the mutant EGFR in glioblastoma, EGFRvIII. In summary, combining EGFR and HDAC inhibitors in glioblastoma may be able to overcome resistance clinically, but the selection of targeted therapies is important to minimize toxicities.

### Bromodomain and extraterminal (BET)

**Fig. 2:**
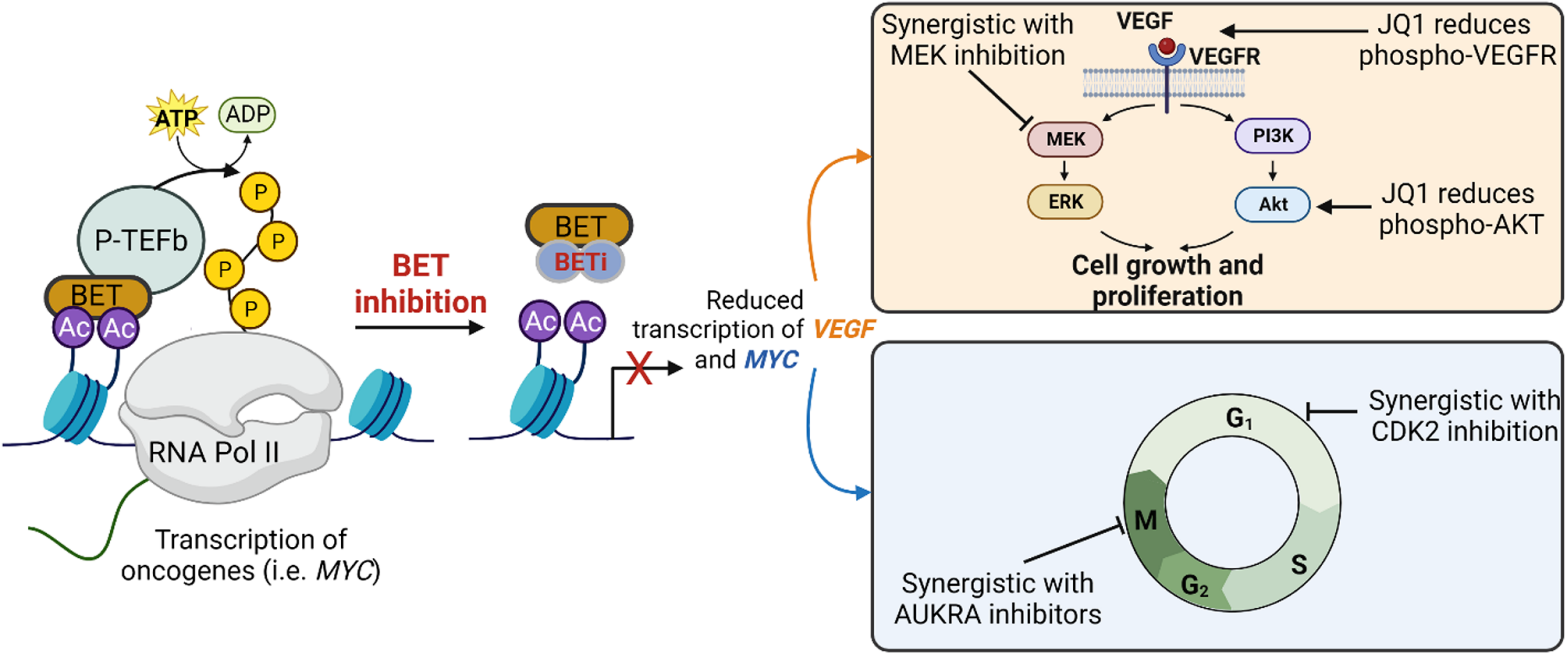
BET Protein Interactions with Key Signaling Pathways in High-Grade Gliomas. BET proteins influence transcriptional processes by recognizing acetylated lysine residues on histones and recruitment of transcriptional factors, including P-TEFb, to activate RNA polymerase [99, 100]. In models of medulloblastoma and HGG, the inhibition of BET proteins impacts the VEGFR/PI3K/AKT signaling pathway [107] and the transcription factor MYC [111]. Furthermore, synergistic effects are observed upon combining BET inhibitors with kinase inhibitors of MEK, AURKA and CDKs, suggesting potential therapeutic strategies [106, 108, 111] Created by BioRender.com

The class of proteins that contain two acetyl-histone reading domains, the bromodomain (BD) and the extraterminal (ET) domain, are collectively known as the bromodomain and extraterminal (BET)-containing proteins. BET proteins are a family of epigenetic readers which recognize specific histone modifications to facilitate the assembly of transcription complexes. This family of epigenetic reader proteins includes BRD2, BRD3, BRD4, and BRDT, and they exert their effects on transcription by binding to acetylated lysine residues on histones [96, 97]. BRD4, perhaps the most studied BET protein, typically recognizes acetylated histone marks on histone 4 lysine 5 and 12 (H4K5 and H4K12) as well as histone 3 lysine 14 (H3K14) [96, 98]. It is proposed that recognizing such acetylated lysine residues can facilitate the activation of transcriptional processes by interacting with cyclinT1 and CDK9 to regulate the positive transcription elongation factor b (P-TEFb) [96, 99, 100]. The BET protein, BRD2, is a protein serine/threonine kinase that promotes the recruitment of transcription factors, such as E2F1, to transcriptional complexes to regulate cell cycle progression [96, 101]. Moreover, BRD2 recognizes acetylated lysine 12 on histone 4 (H4K12) [96, 102]. Several BET proteins, BRD4 and BRD2, have been shown to be overexpressed in gliomas making their inhibition an emerging therapeutic strategy [27]. Unfortunately, resistance to BET inhibition has already been reported secondary to the amplification of RTKs or activation of PI3K/AKT and MAPK signaling pathways [103, 104]. Not only can kinase activation act as a resistance mechanism for BET inhibition, but BET proteins also rewire various kinase transduction pathways to dampen their activity (Fig. 2). A critical understanding of how BET interacts with individual signaling pathways will be important to guide the design and development of novel therapeutic regimens targeting epigenetic readers in HGG.

Past studies have discovered that inhibition of BET proteins can be used to overcome adaptive resistance associated with inhibition of the kinase signaling protein, MAPK kinase (MEK) [105]. While this study was conducted in triple negative breast cancer models, the relationship between BET and MEK in HGG is poorly understood. In glioblastoma models, hexamethylene bisacetamide (HMBA), a high-affinity BRD2 inhibitor, was shown to block cellular proliferation without leading to cell death [106]. Further in vitro and in vivo screenings revealed a synergistic relationship between the combination of HMBA and MEK inhibition leading to enhanced apoptosis of glioma cells [106]. The study concluded that combined inhibition was more effective relative to monotherapy in glioblastoma models and such evidence provides rationale for a clinical trial in selected patients [106]. Moreover, these synergistic effects underscore the convergence of BET proteins with MAPK signaling among malignant brain tumors.

Similar to BET’s influence on MAPK kinases, BET inhibitors also interact with the PI3K/AKT signaling pathways in HGG models to elicit anti-tumor effects. One study used the bromodomain inhibitor, JQ1, to prevent BET protein binding and activity, allowing for the impact of BRD4 in glioma stem cells to be investigated [107]. In this study, JQ1 inhibited cell proliferation, induced cell cycle arrest, and promoted glioma differentiation. The PI3K/AKT signaling pathway mediated the effect of JQ1, and upon inhibition of BRD4, there was a decrease in phosphorylated AKT [107]. This effect was found to be altered upstream via the RTK, vascular endothelial growth factor receptor (VEGFR), where JQ1 inhibited the expression of VEGF and phosphorylated VEGFR [107]. Importantly, JQ1 is a pan-BET inhibitor, and it is likely that BRD4, or a different BET protein, may regulate the growth and development of gliomas via the VEGF/PI3K/AKT signaling axis. Interestingly, another BET inhibitor, OTX015 (birabresib), a BRD3 selective inhibitor, was found to activate the AKT/mTOR pathway by increasing the level of *SESN3*, a protein coding gene for stress-inducible protein, sestrin 3 [77]. The study then combined the treatment of birabresib and everolimus producing additive anti-tumor activity. These studies highlight the role of the BET proteins in the regulation of kinases and suggest improved treatment outcomes with the combination of BET and kinase inhibitors.

Not only do BET proteins cooperate with MAPK and PI3K/AKT signaling cascades, but new studies show that BET proteins interact with kinases involved in mitosis. For example, a recent study reported a synergistic relationship between inhibitors of Aurora Kinase A (AURKA) and BET proteins in MYC-driven glioblastoma cells [108]. AURKA plays a vital role in cell division, in particular during mitosis, and the proper functioning of microtubules [108, 109]. Notably, the oncogenic *MYC* genes are known to be epigenetically regulated by BRD4 via binding at the *MYC* gene promoter region [108, 110]. As expected, the inhibition of BET with the small molecule inhibitor, JQ1, suppressed the expression of *MYCN* in the sensitive cell line [108]. Additionally, the expression of *MYCN* correlated with AURKA levels. Next, JQ1-sensitive and resistant glioblastoma cells displayed a synergistic effect when JQI was combined with an AURKA inhibitor [108]. Ultimately, the discovered relationship between BRD4 and AUKRA inhibitors identified a potential therapeutic approach when translating BET inhibitors into the clinic for trials in HGG with MYC dysregulation.

Cell cycle progression is a fine-tuned biological process with several components essential for its regulation. In addition to kinases like AURKA, cyclin-dependent kinases (CDKs) are required for progression in the cell cycle, and epigenetic interactions may influence these CDKs, creating new opportunities for combination treatments. For example, in MYC-driven models of medulloblastoma, BET inhibition, via JQ1, in combination with CDK inhibitor, milciclib, diminished proliferative markers and induced apoptosis [111]. Importantly, the regulation of MYC via phosphorylation is an essential role of CDK proteins, specifically CDK1 and CDK2 [111–113]. While milciclib suppressed phosphorylation of MYC at residues S62 and T58 to destabilize MYC, BET inhibition reduced *MYC* transcription [111]. The study identified a synergistic relationship between BET and CDK, which reduced medulloblastoma tumor burden and prolonged in vivo survival [111]. In conclusion, combining BET and CDK2 inhibition offers a potential strategic therapy for targeting MYC-dependent medulloblastoma among pediatric patients.

## Epigenetic modulators of histone methylation interplay with kinase signaling cascades

### Protein arginine methyltransferases (PRMT)

**Fig. 3:**
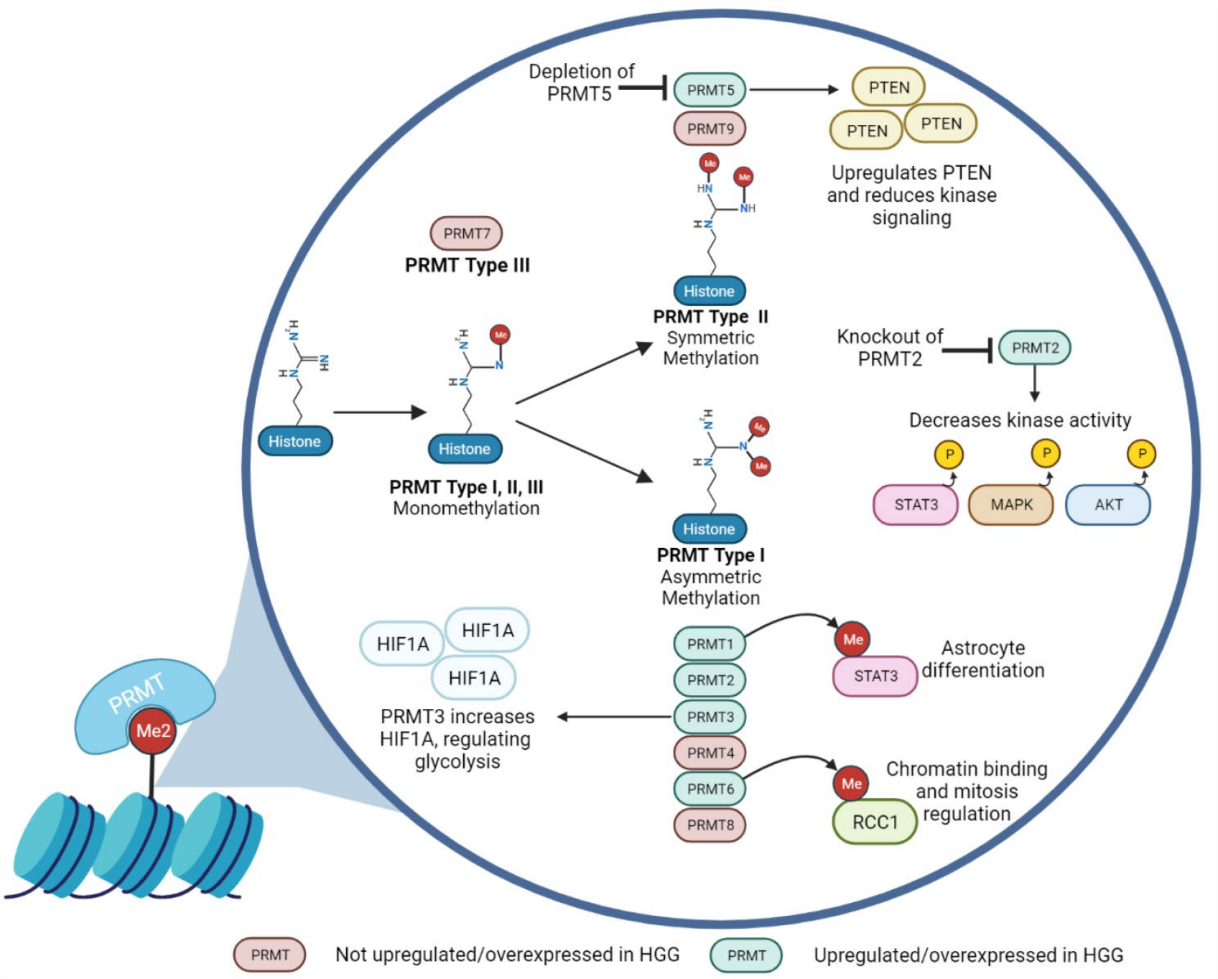
Patterns of PRMT Methylation and Kinase Signaling Crosstalk. The PRMT family initiates distinct methylation patterns, including monomethylarginines, asymmetric dimethylarginines, and symmetric dimethylarginines, to epigenetically regulate cell processes. Highlighted are PRMT1, 2, 3, 5, and 6 which are associated with glioblastoma and medulloblastoma progression and/or tumorigenesis [30, 31, 51, 52, 118]. Functional genetic studies of PRMTs emphasize their modulation of kinase networks. For example, gene depletion of either PRMT2 or 5 is shown to decrease kinase signaling of both PI3K/AKT and MAPK pathways [31, 120]. Moreover, PRMT1 activates JAK/STAT3 pathway to promote cell differentiation, offering insights into potential PRMT-directed therapies [121] Created by BioRender.com

The protein arginine methyltransferase (PRMT) family consists of nine members that act as a component of complexes that epigenetically regulate transcription, translation, splicing, and cell signaling [114, 115]. PRMTs catalyze the transfer of a methyl group to the guanidine nitrogen atoms of arginine, mainly those present on the histone tail. The methylation pattern on arginine by PRMTs can occur in three forms: monomethylarginines, asymmetric dimethylarginines, and symmetric dimethylarginines. Based on the methylation pattern, PRMT members are separated into one of three types. Type I PRMTs form the monomethylarginine and asymmetric dimethylarginine. The type II isoforms produce monomethylarginine and symmetric dimethylarginine. In contrast, type III arginine methyltransferase forms only the monomethylarginine. While mutations in PRMT are uncommon in cancer, high protein expression levels have been associated with poor outcomes [116, 117]. Additionally, PRMT1 and PRMT5 expression has been associated with the development of glioblastoma and medulloblastoma [30, 118]. Overexpression of PRMT3 promotes tumor growth in GBM while also conflicting poor survival with heightened expression; PRMT3 promotes tumorigenesis in GBM by regulating glycolysis, specifically, HIF1A [51]. PRMT2, another overexpressed PRMT protein, confers poor patient prognosis, and knockout of PRMT2 causes reductions of phosphorylated STAT3, AKT, and MAPK in glioma cell lines [31]. Meanwhile, PRMT6 is overexpressed in GBM, causing increased self-renewal, and PRMT6 expression is correlated to poor patient prognosis [52]. PRMT6 is postulated to methylate RCC1 (regulator of chromosome condensation 1) for chromatin binding, thereby modulating mitosis [51, 52]. Therefore, several family members of PRMT have recently become of interest as cancer targets based on the association of PRMT expression with poor patient outcomes and brain development and tumorigenesis (Fig. 3). Numerous PRMT inhibitors have been developed and are largely separated into Type I PRMT and Type II PRMT inhibitors which have been investigated in various cancer models [119]. Drug discovery efforts are ongoing to design new agents with selective activity against different isoforms of PRMT and considerations for strategies to improve efficacy through combination treatments.

The PRMT-PTEN signaling axis is one potential intersection for consideration when designing combination treatments with PRMT inhibitors. As a tumor suppressor, PTEN attenuates the kinase signaling cascade of the PI3K/AKT/mTOR pathway. In glioblastoma models, one study found *PTEN* as a downstream target of the type II arginine methyltransferase, PRMT5 [120]. Here, PRMT5 expression was enriched at the promotor region of *PTEN* in glioblastoma neurospheres but not in the differentiated glioblastoma counterpart. Depletion of PRMT5 caused the expression of PTEN transcript and protein expression to significantly increase in the glioblastoma neurospheres [120]. In parallel, the expression of phosphorylated AKT and ERK was reduced with gene silencing of PRMT5. Furthermore, reduced proliferative and self-renewal capacity among the neurospheres was observed following PRMT5 depletion, partially restored upon *PTEN* knockdown. Overall, this study demonstrates *PTEN* as a target of PRMT5 methylation, which regulates important processes involving cell proliferation, cell growth, and self-renewal of neurospheres.

Like the previously described histone targets, PRMTs can also have non-histone targets. The type I arginine methyltransferase, PRMT1, influences the JAK/STAT3 pathway in neural stem precursor cells. Activated STAT3 signaling can impact many cellular processes, including cell differentiation. Activation of STAT3 can be achieved via phosphorylation or methylation. A recent study found STAT3 as a non-histone target of PRMT1, and its methylation resulted in the enhanced activation of STAT3 [121]. The methylated STAT3 promoted astrocytic differentiation of neural stem precursor cells [121]. Taken together, this study demonstrates STAT3 regulation via PRMT1 to promote cell differentiation. This is relevant to HGG when constructing treatment strategies to target PRMT1 pharmacologically, leading to therapies that will promote differentiation of stem-like cancer cells.

### Enhancer of zeste homolog 2 (EZH2)

Enhancer of zeste homolog 2 (EZH2) is an epigenetic writer that catalyzes the methylation of lysine residues on histone and non-histone targets through its involvement with Polycomb-group (PcG) proteins. EZH2 performs a critical role in transcription processes, particularly transcriptional repression, by acting as the functional subunit of the PcG protein complex, Polycomb Repressive Complex 2 (PCR2) [122, 123]. The methyltransferase activity of EZH2 arises from its SET domain and uses S-adenosyl-L-methionine (SAM) as a cofactor [124]. The PRC2 houses the core proteins: EZH1/2, Suz12, Eed, and Rbbp4 [125]. Collectively, the primary target of PRC2 is histone H3 lysine 27 (H3K27), which recruits PRC1 to exert alterations in gene expression via chromatin remodeling [124]. Interestingly, EZH2 has demonstrated a potential tumor-suppressive role in subsets of diffuse midline gliomas by inducing oxidative phosphorylation [126]. However, a different study identified EZH2 inhibition as a therapeutic target among gliomas harboring H3K27M mutations, which inhibits the PRC2 complex [127]. In glioblastoma, high expression of EZH2 has been associated with worse survival and high tumor grade [33]. Based on the proposed role of EZH2 in glioblastoma, inhibition of this writer has triggered interest as a therapeutic target [128]. While preclinical studies are ongoing, caution is necessary when considering using EZH2 inhibitors in other glioma models, as EZH2 may have context-specific functions and various downstream effects such as regulation of tumor suppressors or other signaling pathways.

**Fig. 4:**
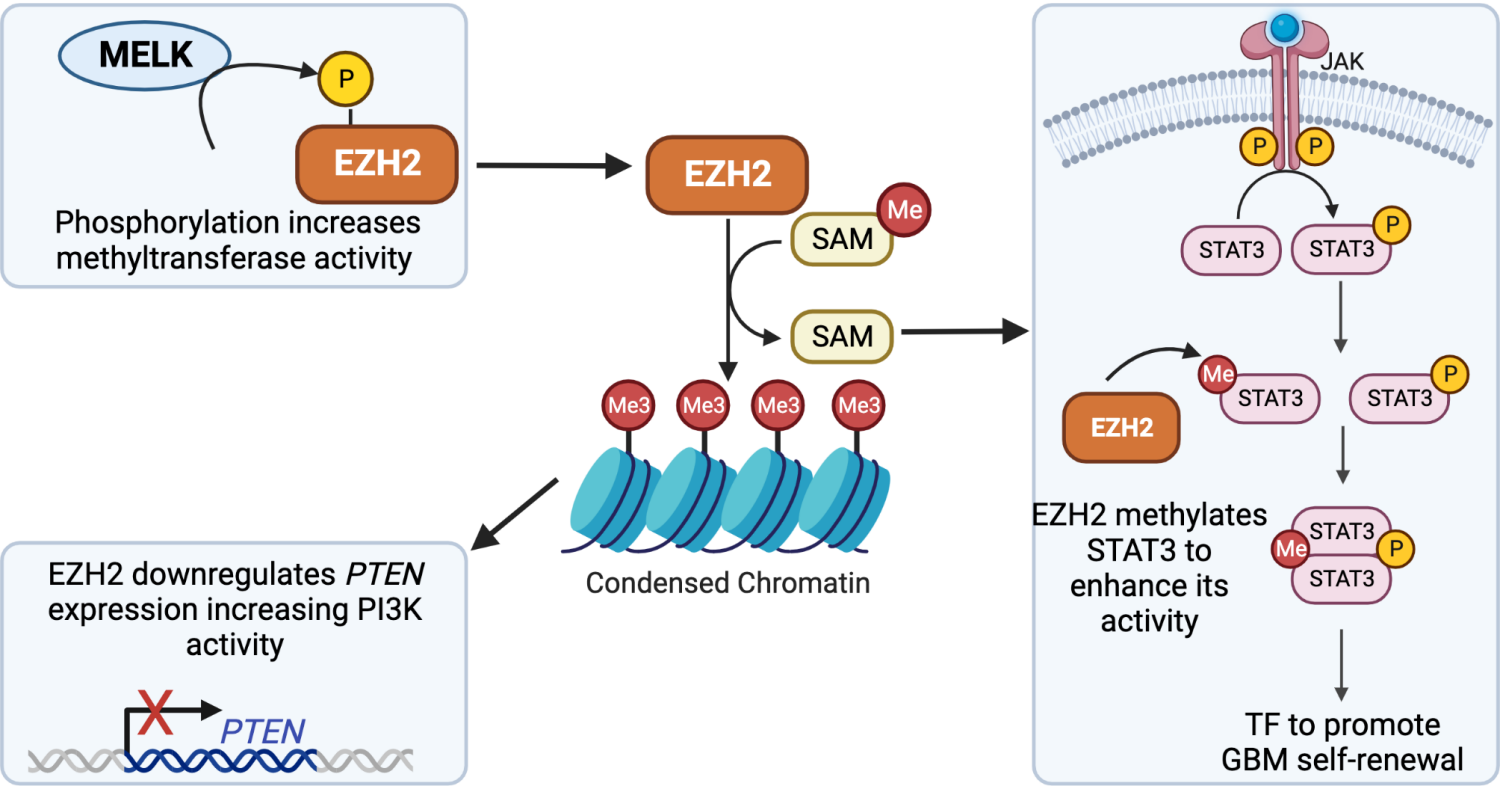
Multifaceted Role of EZH2 in Kinase-Mediated Regulation. As a methyltransferase, EZH2 facilitates the compaction of chromatin by adding methyl groups to histone protein, H3K27. This epigenetic modification condenses the chromatin structure, affecting gene expression patterns. In this manner, EZH2 can exert its influence on kinase signaling pathways. It downregulates *PTEN* expression, leading to activation of the PI3K/AKT pathway [129]. Beyond its chromatin-related functions, EZH2 acts as a methyltransferase for non-histone protein, STAT3, increasing its activity in gene regulation [134]. Moreover, the kinase MELK plays a crucial role, as it phosphorylates EZH2, enhancing its methyltransferase activity and amplifying its effect [131, 133]. TF – transcription factor Created by BioRender.com

In an effort to develop EZH2 inhibitors, several small molecules have been designed to prevent its activity with varying mechanisms of action [122]. Current EZH2 inhibitors that are in development include SAM-competitive inhibitors, inhibitors that disrupt EZH2 protein interactions, and those that promote EZH2 degradation [122]. One of the largest groups of inhibitors is those that bind to the SET domain and compete with SAM to inhibit the methyltransferase activity of EZH2, such as tazemetostat and GSK126. Similarly, there is a drug in development, 3-Deazaneplanocin A (DZNep), which inhibits global histone methylation by targeting S-adenosyl homocysteine (SAH) hydrolase. Another group of EHZ2 inhibitors work by disrupting EZH2s’ interaction with the PCR2 complex. These inhibitors target the PCR2 scaffolding proteins, Suz12 and Eed, and include A769662 and astemizole, respectively. Lastly, there is a class of EZH2 inhibitors that promote its degradation, including ANCR, GNA002, and FBW7. In addition to direct inhibition of EZH2, it is important to consider other means of vulnerability, perhaps through its interactions with kinase signaling pathways including the PI3K/AKT and JAK/STAT pathways (Fig. 4).

Evaluation of EZH2 and its relationship with cell signaling processes may provide insights into kinase signaling dysfunction or mechanisms of therapeutic resistance. EZH2’s cellular interplay with E2F transcription factors in glioblastoma models is one mechanism that offers tumor cell proliferation and growth. The clinically relevant E2F transcription factor, E2F7, was overexpressed among high-grade glioma patients compared with low-grade gliomas or normal tissues. Furthermore, high expression of E2F7 was associated with poor prognosis [129]. Moreover, E2F7 acts upstream of EZH2 as a transcriptional activator in glioblastoma by binding to its promoter [129]. Kinase signaling pathways are affected by the activity of E2F7 and its action on EZH2. The tumor suppressor protein, PTEN, is a negative regulator of the PI3K/AKT signaling. PTEN, an established target of EZH2, is downregulated in glioblastoma and associated with poor survival [129]. Overall, E2F7 is responsible, in part, for the proliferative advantages in glioblastoma mediated by EZH2 inhibition of PTEN and leading to activation of the PI3K/AKT/mTOR pathway [129]. The relationship between EZH2 and PTEN highlights critical insights into the origins of aberrant signaling in glioblastoma and considerations for therapy.

Interestingly, the PTEN/PI3K/AKT signaling network is not the only kinase signaling cascade EZH2 interacts with among brain tumor models. EZH2 interacts with maternal embryonic leucine-zipper kinases (MELK) in glioblastoma and medulloblastoma. MELK is an important kinase signaling protein involved in cell growth, cell cycle regulation, DNA repair, migration, invasion, and apoptosis [130–132]. In glioblastoma, poorer overall survival was noted among patients with higher expression levels of MELK and EZH2 [131]. Similar to glioblastoma, medulloblastoma patients had poor overall survival with increased MELK expression postoperatively [133]. Beyond its association with poor prognosis, MELK activates EZH2 through phosphorylation to promote glioma stem-like cells to proliferate and self-renew [131]. EZH2 and MELK also have a similar relationship in medulloblastoma. In medulloblastoma models, phosphorylated EZH2, and its H3K27 methylation activity, were reduced upon MELK gene silencing [133]. Importantly, in vivo models of medulloblastoma displayed a significant survival benefit from the knockdown and pharmacological inhibition of MELK and EZH2, highlighting the relevance of these two proteins in brain tumor proliferation [133]. Once EZH2 is activated via phosphorylation in glioblastoma, it proceeds to methylate a downstream target, NFĸB. NFĸB methylation mediates the effect of EZH2 to induce glioblastoma proliferation and maintenance of stem-like characteristics [131]. Nonetheless, EZH2 and MELK cooperate in glioblastoma and medulloblastoma to promote cell proliferation and this interplay offers a potential combination therapeutic strategy.

The interactions between EZH2 and its non-histone targets are also important in HGG, and this relationship could offer further understanding of oncogenic signaling pathways. One non-histone target of EZH2 is the STAT3 in the JAK/STAT pathway [134]. Following JAK activation by interleukin, interferons, or growth factors, STAT3 is phosphorylated and acts as a transcription factor to promote various processes such as immune cell response, cell division, metastasis, and cell differentiation [135]. Post-translational modifications of STAT3, such as methylation, can affect its activity. Upon methylation of STAT3 via EZH2 in glioblastoma stem-like cells, STAT3 activity was enhanced [134]. This positive regulation promoted the self-renewal capacity of stem-like glioblastoma cells. As expected, EZH2 inhibition via gene knockdown and pharmacological inhibition, via DZNep, reduced the methylation of STAT3 and dampened its activity [134]. Ultimately, EZH2 directly regulates STAT3, and its inhibition may impair key signaling pathways that promote tumor growth and maintain a stem-like tumor cell population in glioblastoma.

### Lysine-specific demethylase 1 (LSD1)

Histone demethylases, including lysine-specific demethylase 1 (LSD1/ KDM1A), regulate gene transcription and chromatin structure via the demethylation of lysine residues. LSD1 was the first discovered lysine demethylase, and it belongs to a family of two histone lysine demethylases (LSD1 and LSD2) [136]. LSD1 is a flavin-dependent monoamine oxidase that catalyzes the demethylation of mono- and dimethyl groups from histone 3 on lysine residues 4 and 9 (H3K4 and H3K9) [136]. Overexpression of LSD1 is found in many cancer types, and the resulting increase in its activity can lead to gene dysregulation and support cancer progression [137]. For example, LSD1 plays a role in cancer by maintaining cancer stemness, regulating differentiation, promoting EMT, and regulating hypoxia [138]. Therefore, LSD1 is regarded as a cancer drug target with several small molecule inhibitors already in various stages of clinical development. In HGG, LSD1-directed agents can induce tumor regression when assessed in vivo [36, 139, 140].

Pharmacological LSD1 inhibitors have been developed and are separated into two categories, reversible and irreversible inhibitors. The first identified irreversible inhibitor was tranylcypromine. Tranylcypromine covalently binds to the FAD domain within the active site of LSD1, rendering LSD1 inactive [141–145]. Since the identification of tranylcypromine, multiple irreversible LSD1 inhibitors have been developed and have been investigated in various other tumor models, mostly hematological and small-cell lung cancer; these molecules include GSK-LSD1, ORY-1001, RN-1, IMG-7289, INCB059872, and ORY-2001 [142, 143, 145–148]. In contrast, reversible LSD1 inhibitors, such as SP-2509, are proposed to bind to the allosteric site and have effects independent of LSD1 demethylase activity [149]. Meanwhile, another reversible inhibitor, CC-990,011 binds at the amine oxidase pocket of LSD1 while having anti-tumor activity in small-cell lung cancer [150].

**Fig. 5:**
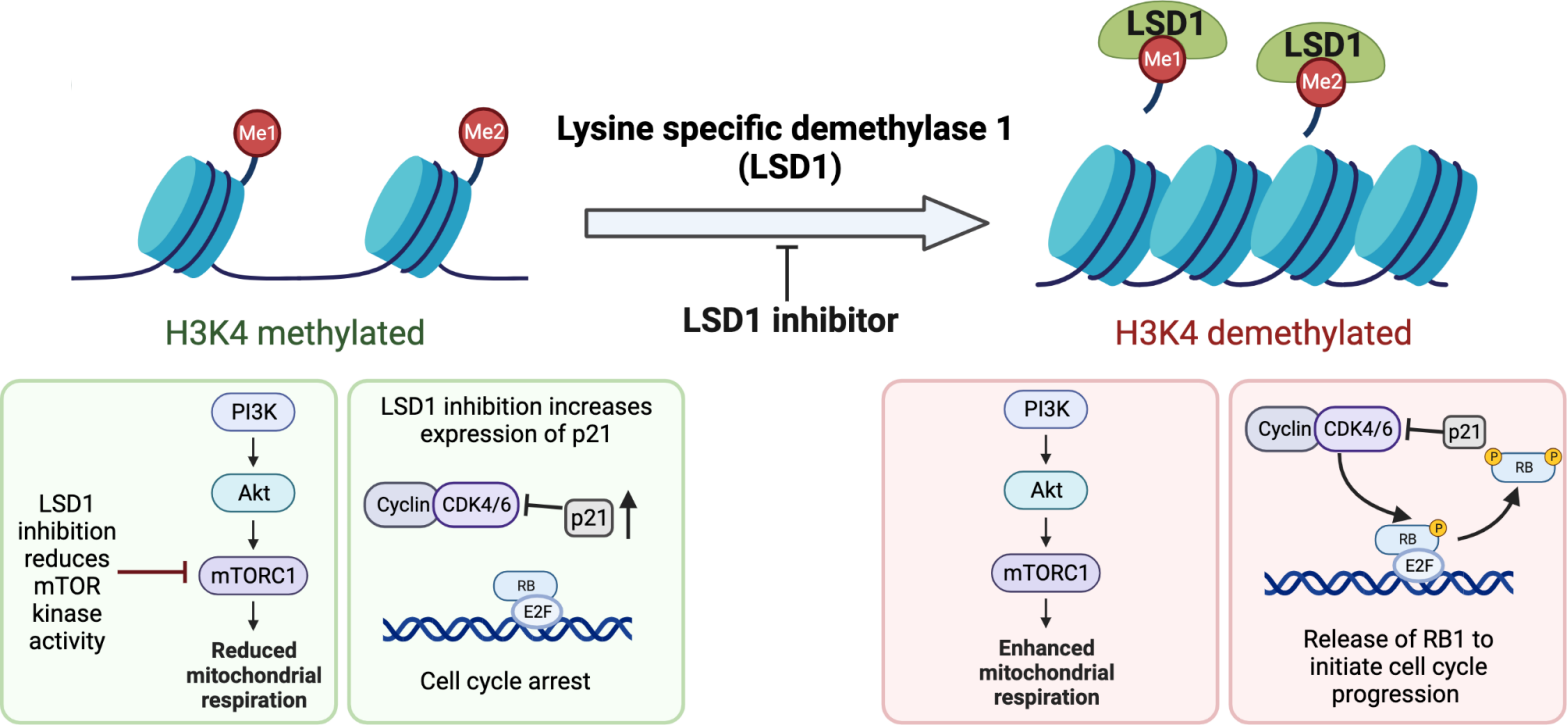
LSD1 Modulation of Cell Growth and Kinase Signaling Pathways. As a histone demethylase, LSD1 targets H3K4 demethylation, influencing crucial cellular processes. Inhibition of LSD1 leads to reduced mTOR signaling and subsequent attenuation of mitochondrial respiration, impacting cell metabolism and growth [151]. Moreover, LSD1 inhibition upregulates the expression of p21, a negative regulator of the cell cycle, culminating in cell cycle arrest [151, 152]. In contrast, cells without LSD1 inhibition demonstrate heightened cell growth and progression through the cell cycle Created by BioRender.com

Part of the mechanism by which LSD1 inhibitors induce an anti-tumor response is through its interaction with kinase signaling pathways involving cell metabolism and cell cycle progression (Fig. 5). One study found that LSD1 inhibition via tranylcypromine impaired mitochondrial respiration in glioblastoma cells by reducing their oxidative capacity [151]. This impairment was accompanied by a reduction in mitochondrial proteins Tom20, PDH, and SDH. Furthermore, tranylcypromine treatment caused a decrease in the kinase activity of mTOR that reduced the downstream activation of two key players to regulate cell growth and cell cycle progression: ribosomal protein S6 kinase beta-1 (S6K1) and eukaryotic translation initiation factor 4E-binding protein 1 (4-EBP1) [151]. Overall, this study concluded that LSD1 inhibition impairs mitochondrial respiration with subsequent effects on cell cycle progression.

The effects of LSD1 inhibition on cell cycle progression extend beyond its influence on mTOR signaling. An additional role for LSD1 inhibitors in glioblastoma is to induce cell senescence through its involvement in the retinoblastoma (RB)/E2F/CDK-Cyclin pathway. Both gene silencing and pharmacological inhibition of LSD1 increased the expression of cyclin-dependent kinase inhibitor, p21 [151]. Accordingly, the upregulation of p21 led to the reduced phosphorylation of RB and a negative cell cycle regulation to induce cell senescence. A different study in glioblastoma also found that LSD1 activity/inhibition regulates the expression of genes involved in cell cycle progression. Following inhibition of LSD1, the gene encoding p21 was upregulated, and its downstream targets, cyclin-dependent kinase (CDK4/6/2), had reduced activity [152]. Interestingly, this study combined LSD1 inhibition, GSK-LSD1, with a small molecule inhibitor that disrupts EZH2 interactions, AC1Q3QWB, with an oncogenic long noncoding RNA (lnRNA), HOTAIR (Hox transcript antisense intergenic RNA). The combination treatment yielded synergistic cell cycle inhibition via upregulation of *CDKN1A* encoding p21 and resulted in improved survival of orthotopic glioblastoma models [152]. In summary, LSD1 expression is implicated in promoting HGG, and its successful inhibition disrupts cell cycle progression through the interplay with kinase signaling networks.

## Discussion

The review of recent studies in HGG and medulloblastoma shows that the interplay between epigenetics and kinase signaling pathways is a multifactorial mechanism. Most likely, the precise relationship has tumor-specific and drug-specific implications. Overall, the cooperation between epigenetic and kinase signaling networks highlights new multimodal treatment strategies to build upon and enhance the standard of care in HGG. As epigenetically directed therapies and kinase inhibitors continue to translate into the clinic, sustaining partial and complete responses to single target agents will likely be challenging. Studies should continue to look for opportunities to understand the interplay of chromatin regulators and kinase signaling networks to overcome single-agent barriers. In addition, studies should evaluate the treatment regimens for kinase/chromatin inhibitors including the timing of treatment, sequence of drug administration, and dosing to maximize efficacy and limit toxicities. Understanding these parameters will be necessary to build novel therapeutic strategies with greater efficacy against HGGs.

Targeted therapies have become a significant tool in treating many cancer types, but their utility in HGG has not yet been established. A major constraint for targeted therapy in HGG is dose-limiting toxicities (DLTs). The DLTs from targeted therapy are a result of off-target and off-tumor effects. For example, most epigenetically targeted inhibitors are pan-inhibitors, which target all or several proteins within that class. Developing more selective agents, including both epigenetic and kinase inhibitors, will aid in reducing off-target effects and improve safety. Off-tumor effects are a consequence of target inhibition outside of the tumor site; this can be opposed by using small molecule inhibitors with high brain penetrance to reach adequate concentrations within the tumor. Another factor to consider is the tumor-specific mechanism and individual genomic landscape that predict response to targeted therapy. This measure is already relevant as EZH2 can act in a tumor-specific manner with oncogenic activity in most tumors, but a tumor suppressive role in a small subset of tumors, including cases of DMGs [126, 153]. Thus, this idea of identifying predictive biomarkers, or precision medicine, by analyzing a patient’s genomic landscape will improve patient selection and spare non-responders from toxicity. Inhibitors need to be more effective at lower doses and given to patients with a high likelihood of a response to improve the toxicity profile of targeted therapy.

Beyond safety, resistance is another obstacle to targeting chromatin modifiers and kinase signaling proteins. Due to the redundancy in kinase signaling pathways, inhibiting a single kinase is often unsuccessful, as compensatory pathways mitigate the effects of single-agent kinase inhibition. An additional mechanism of resistance to kinase inhibition is through epigenetic changes. Furthermore, epigenetic regulation is highly interdependent between the writers, readers, and erasers and can produce unexpected effects that may limit therapeutic efficacy. For example, inhibition of HDAC via vorinostat can increase H3K4 methylation, making it vulnerable to LSD1 demethylation [35]. One solution to overcome resistance and improve drug toxicity profile is to design combination treatment strategies inclusive of kinase inhibition and epigenetically directed inhibition.

Several in vitro and in vivo models have identified synergistic relationships between kinase and epigenetic inhibition. However, there is still a need to explore the interplay between epigenetic regulators and kinase signaling pathways and understand their specific mechanisms. Identifying safe drug combinations with synergistic or additive effects may afford improvements in efficacy and create an opportunity in the clinical trial setting for HGGs. In the clinic, treatment combinations could allow for dose reductions that exert a clinical effect and decrease unwanted side effects. Certain drug combinations may also circumvent resistance mechanisms associated with single-agent inhibition to extend a drug response. Additionally, other combination treatment strategies to overcome the lack of single-agent success may include introducing immunotherapies to either kinase inhibition or epigenetically directed agents. Previous studies have shown that epigenetic alterations change the tumor microenvironment to contribute to the immune suppressive niche for tumor cells. More recently, studies in several cancer types have shown inhibition of epigenetic regulators (LSD1, EZH2, BET proteins) can enhance the anti-tumor immune response of anti-PD1 therapy [13, 154–156]. Interestingly, studies in other cancer models highlight that kinase inhibition can enhance anti-tumor effects with immunotherapies [157–159]. Investigating triple therapy targeting chromatin modulators and kinase pathways and using immunotherapy in the ongoing efforts to attenuate tumor cell proliferation to improve patient outcomes and increase overall survivability would be worthwhile. In conclusion, enhancing our understanding of the cooperation across the HGG epigenome and genome will guide the development of new therapeutic strategies.

## Data Availability

Not applicable.

## Nomenclature

4-EBP1: Eukaryotic translation initiation factor 4E-binding protein 1
AMP: Amplification
AURKA: Aurora kinase A
BBB: Blood-brain-barrier
BET: Bromodomain and extraterminal
BRD) c-Jun N-terminal kinase (JNK: Bromodomain
c-MET: Mesenchymal-epithelial transition factor
CDKN2A: Cyclin-dependent kinase inhibitor 2 A
CDKs: Cyclin-dependent kinases
CNA: Copy number alterations
CNS: Central nervous system
CoREST: REST corepressor 1
DCR: Disease control rate
DIPG: Diffuse intrinsic pontine glioma
DLT: Dose-limiting toxicity
DMG: Diffuse midline gliomas
DNMT: DNA methyltransferase
DZNep: 3-Deazaneplanocin A
EGFR: Epidermal growth factor receptor
ERK: Extracellular signal-regulated kinase
EZH2: Enhancer of zeste homolog 2
FGFR: Fibroblast growth factor receptor
FOXM1: Forkhead box M1
G-CIMP: Glioma – CpG island methylator phenotype
GBM: Glioblastoma
H3K4/9/27: Histone 3 lysine 4/9/27
H4K5/8/12: Histone 4 lysine 5/8/12
HATs: Histone acetyltransferase
HDAC: Histone deacetylase
HGGs: High-grade gliomas
HMBA: Hexamethylene bisacetamide
HOMDEL: Homozygous deletion
HOTAIR: Hox transcript antisense intergenic RNA HOTAIR
IDH: Isocitrate dehydrogenase
JAK: Janus kinase
KIT: Mast/stem cell growth factor receptor
lnRNA: Long noncoding RNA
LSD1/KDM1A: Lysine-specific demethylase 1
MAPK: Mitogen-activated protein kinase
MEK: Mitogen-activated protein kinase kinase
MELK: Maternal embryonic leucine-zipper kinases
MGMT: O6-methylguanine-DNA methyltransferase
MKP1: MAPK phosphatase 1
MTD: Maximum tolerated dose
mTOR: Mammalian target of rapamycin
NF1: Neurofibromin 1
NuRD: Nucleosome remodeling and deacetylase
ORR: Overall response rate
OS: Overall survival
P-TEFb: Positive transcription elongation factor b
PcG: Polycomb-group
PD: Pharmacodynamic
PDGFRA: Platelet-derived growth factor receptor
PFS: Progression-free survival
pHGG: Pediatric high-grade glioma
PI3K: Phosphatidylinositol-3 kinase
PK: Pharmacokinetic
PRC2: Polycomb repressive complex
PRMT: Protein arginine methyltransferase
PTEN: Phosphatase and tensin homolog
RAS: Rat sarcoma virus
RB: Retinoblastoma
RCC1: Regulator of chromosome condensation 1
RT: Radiation therapy
RTKs: Receptor tyrosine kinases
S6K1: S6 kinase beta-1
SAH: S-adenosyl homocysteine
SAM: S-adenosyl-L-methionine
STAT: Signal transducer and activator of transcription
VEGFR: Vascular endothelial growth factor receptor
WHO: World Health Organization
